# Draft genome data of *Pediococcus acidilactici* strain AP1 isolated from common carp (*Cyprinus carpio*)

**DOI:** 10.1016/j.dib.2026.113296

**Published:** 2026-09-28

**Authors:** Fazliddin Kobilov, Nilufar Elova, Bobokulov Murodali, Bekmurodova Gullola, Dildora Amirsaidova, Mastura Xidirova, Farangiz Yodgorova, Shakhlo Miralimova

**Affiliations:** a Laboratory of Human Microbiome, Institute of Microbiology of the Academy of Science of the Republic of Uzbekistan, Uzbekistan; b Institute of Microbiology of the Academy of Science of the Republic of Uzbekistan, Uzbekistan

**Keywords:** Pediococcus acidilactici, Whole-genome sequencing (WGS), Lactic acid bacteria, Common carp, Aquaculture microbiology, Phylogenomics

## Abstract

This data article presents the whole-genome sequencing and associated genomic datasets of *Pediococcus acidilactici* strain AP1, isolated from the intestinal tract of a healthy common carp (*Cyprinus carpio*) obtained from a fish farm in the Tashkent region, Uzbekistan. The datasets comprise raw paired-end sequencing reads, a draft genome assembly, genome-quality assessment reports, structural and functional annotations, and phylogenomic outputs. Whole-genome sequencing was performed using the Illumina MiSeq i100 Plus System with paired-end 2 × 151 bp chemistry. De novo assembly generated a draft genome comprising 19 contigs, with a total length of 2,015,983 bp, an N50 of 388,737 bp, an L50 of 2, and a GC content of 42.02%. CheckM2 estimated 99.99% genome completeness and 0.5% contamination, whereas BUSCO recovered 99.2% of the conserved bacterial single-copy orthologs. Annotation with Prokka v1.15.6 identified 1,897 protein-coding sequences, 52 tRNA genes, four rRNA genes, and one tmRNA. Genome-based phylogenetic and digital DNA–DNA hybridization analyses supported the assignment of strain AP1 to P. acidilactici. Functional annotations generated using Prokka, RAST, eggNOG-mapper, and InterProScan indicated genes putatively associated with carbohydrate fermentation, stress adaptation, exopolysaccharide biosynthesis, peptide transport and proteolysis, cell-surface interactions, and CRISPR–Cas defense. Gene-based functional assignments represent predictions and require experimental validation. The publicly available datasets can support comparative genomic investigations of fish-associated *Pediococcus* strains and future experimental studies of host-associated and probiotic-relevant traits.

Specifications Table

**Table:** 

Subject	Microbiology
Specific subject area	Microbiology; microbial genomics; aquaculture microbiology
Type of data	Raw sequencing reads; genome assembly; annotation files; tables; figures; phylogenomic data
Data collection	Genomic DNA extracted from a pure culture of *Pediococcus acidilactici* strain AP1 was sequenced using the Illumina MiSeq i100 Plus platform with paired-end 2 × 151 bp chemistry. Raw reads were assessed using FastQC v0.12.1, filtered using fastp v1.1.0, and assembled de novo using SPAdes v4.2.0
Data format	Raw and filtered: FASTQ.GZ; analyzed: FASTA, GFF3, GBK, FAA, FFN, TSV, JSON, HTML, Newick and PDF
Description of data collection	Strain AP1 was isolated from the intestinal tract of a healthy common carp collected from a fish farm in the Tashkent region, Uzbekistan. The dataset includes raw reads, genome assembly, quality-control reports, structural and functional annotations, and phylogenomic outputs.
Data source location	Institution: Institute of Microbiology, Academy of Sciences of the Republic of Uzbekistan; City/Region: Tashkent; Country: Uzbekistan; Biological source: intestinal tract of common carp (*Cyprinus carpio*)
Data accessibility	National Center for Biotechnology Information (NCBI) GenBank Direct URL to data: https://www.ncbi.nlm.nih.gov/bioproject/1476265 https://www.ncbi.nlm.nih.gov/biosample/SAMN60696776 https://doi.org/10.17632/WJDNTC39SP.1
BioProject	PRJNA1476265
BioSample	SAMN60696776
SRA accession	SRR39936145
Direct URL	https://www.ncbi.nlm.nih.gov/sra/SRR39936145
Mendeley Data	DOI: https://doi.org/10.17632/WJDNTC39SP.1
Assembly accession	GCA_058312935.1
Whole Genome Shotgun accession	JBZEGU000000000

## 1 Value of the Data

• This dataset expands the publicly available genomic resources for fish-associated lactic acid bacteria and broadens the geographic representation of *Pediococcus acidilactici* genomes by providing raw sequencing reads, a draft genome assembly, genome annotations, quality-control reports, and phylogenomic outputs for strain AP1 isolated from common carp in Uzbekistan, Central Asia.
• Researchers in microbial genomics, aquaculture microbiology, lactic acid bacteria, and probiotic research can use these data as a reference for studying the genomic diversity of fish-associated *Pediococcus* strains.
• The genome sequence and accompanying annotation files can be reused in comparative genomics, pangenome analysis, phylogenomics, ortholog identification, taxonomic assessment, and the design of strain-specific molecular experiments.
• The annotated genome contains candidate genes putatively associated with carbohydrate fermentation, stress adaptation, exopolysaccharide biosynthesis, proteolytic systems, surface-associated functions, and CRISPR–Cas defense, which can guide subsequent experimental validation.
• The availability of both raw and processed data enables independent reanalysis, assessment of alternative assembly and annotation workflows, and comparison with newly sequenced *Pediococcus* genomes.

## 2 Background

*Pediococcus acidilactici* is a Gram-positive, facultatively anaerobic lactic acid bacterium belonging to the family *Lactobacillaceae*. Members of this species are frequently isolated from fermented foods and the gastrointestinal tracts of humans and animals, including fish, and contribute to carbohydrate fermentation through lactic acid production [[1], [2], [3]]. Owing to these characteristics, selected *P. acidilactici* strains have attracted interest for potential applications in food biotechnology, animal production, and aquaculture [3,4].

The use of probiotic microorganisms is increasingly being investigated as a strategy to support fish health and reduce reliance on antibiotics in aquaculture. Previous studies have shown that selected *Pediococcus* strains can modulate intestinal microbial communities, improve nutrient utilization, influence immune responses, and enhance resistance to environmental stress and infectious diseases in cultured fish [[4], [5], [6]]. These observations have encouraged genomic characterization of fish-associated *Pediococcus* isolates to investigate their genetic diversity and identify candidate functions for subsequent experimental validation.

This data article presents the draft genome sequence of *P. acidilactici* strain AP1, isolated from the intestinal tract of a healthy common carp (*Cyprinus carpio*) collected from a fish farm in the Tashkent region, Uzbekistan. The dataset includes raw sequencing reads, a draft genome assembly, quality-assessment reports, structural and functional annotations, and phylogenomic outputs. These publicly available data provide a valuable resource for comparative genomic analyses and future investigations of genome-encoded functions potentially associated with host adaptation and probiotic-related traits.

## 3 Data Description

The dataset contains raw paired-end sequencing reads, a draft genome assembly, genome-quality assessment reports, structural and functional annotation files, and phylogenomic outputs for *P. acidilactici* strain AP1, isolated from the intestinal tract of a healthy common carp (*C. carpio*) collected from a fish farm in the Tashkent region, Uzbekistan. The raw sequencing reads are available through the NCBI Sequence Read Archive under accession SRR39936145. The draft genome assembly is available in NCBI GenBank under Assembly accession GCA_058312935.1 and WGS accession JBZEGU000000000. The complete quality-control, annotation, and phylogenomic output files, together with the source data underlying Fig. 1, Fig. 2—including the Proksee CGView.js project JSON file and the GBDP intergenomic distance matrix—are available through Mendeley Data [7].

**Fig. 1:**
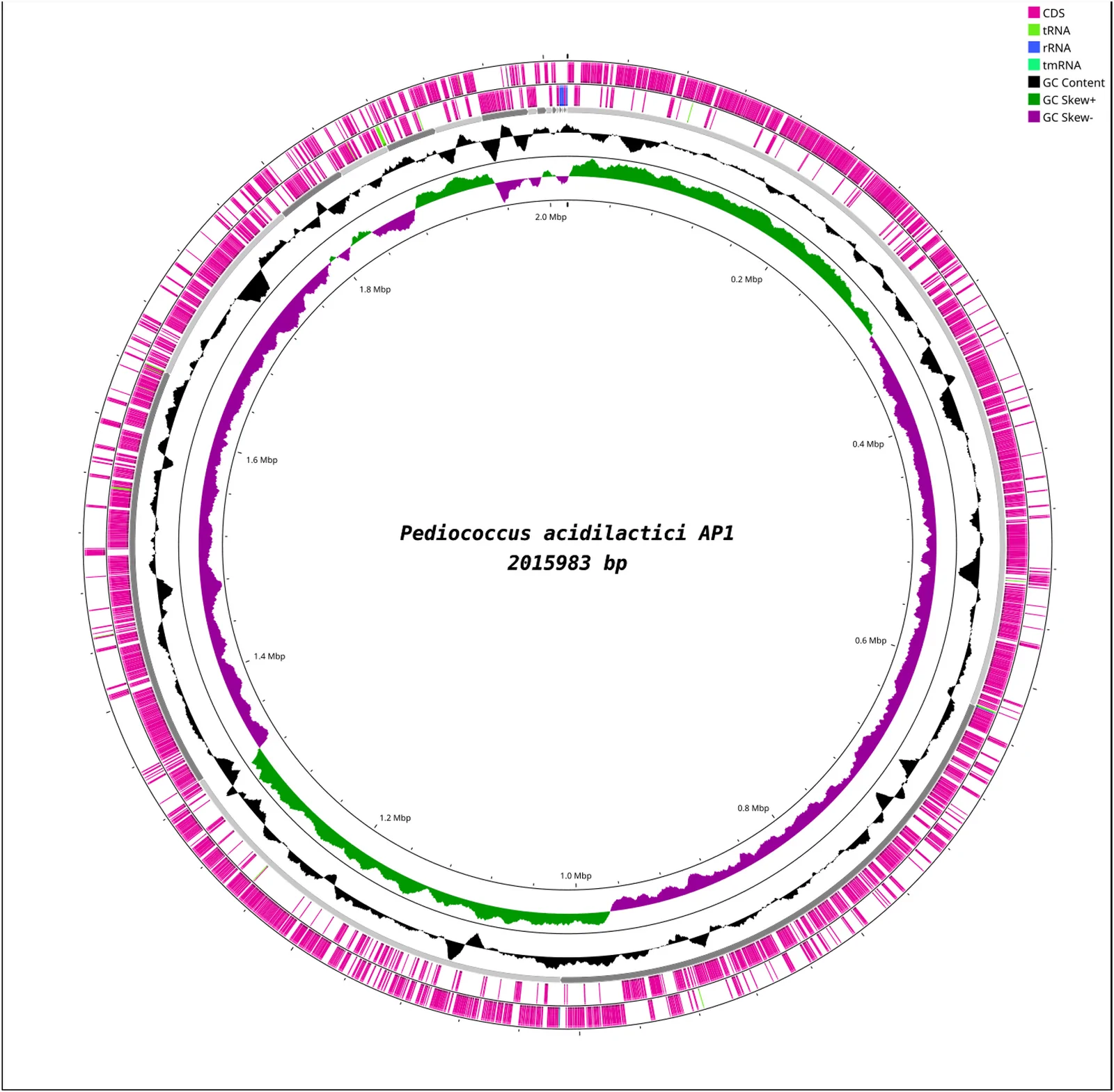
Circular visualization of the draft genome assembly of *Pediococcus acidilactici* strain AP1. The map was generated using Proksee v1.1.0 from the 19-contig draft genome assembly. The displayed tracks represent protein-coding sequences located on the forward and reverse strands, tRNA, rRNA, and tmRNA genes, GC content, and GC skew. Positive and negative GC-skew values are shown in green and purple, respectively. Genome coordinates are displayed in megabase pairs, and the total assembly length is 2,015,983 bp. The circular layout is used for visualization and does not indicate that the chromosome was assembled as a closed circular replicon.

**Fig. 2:**
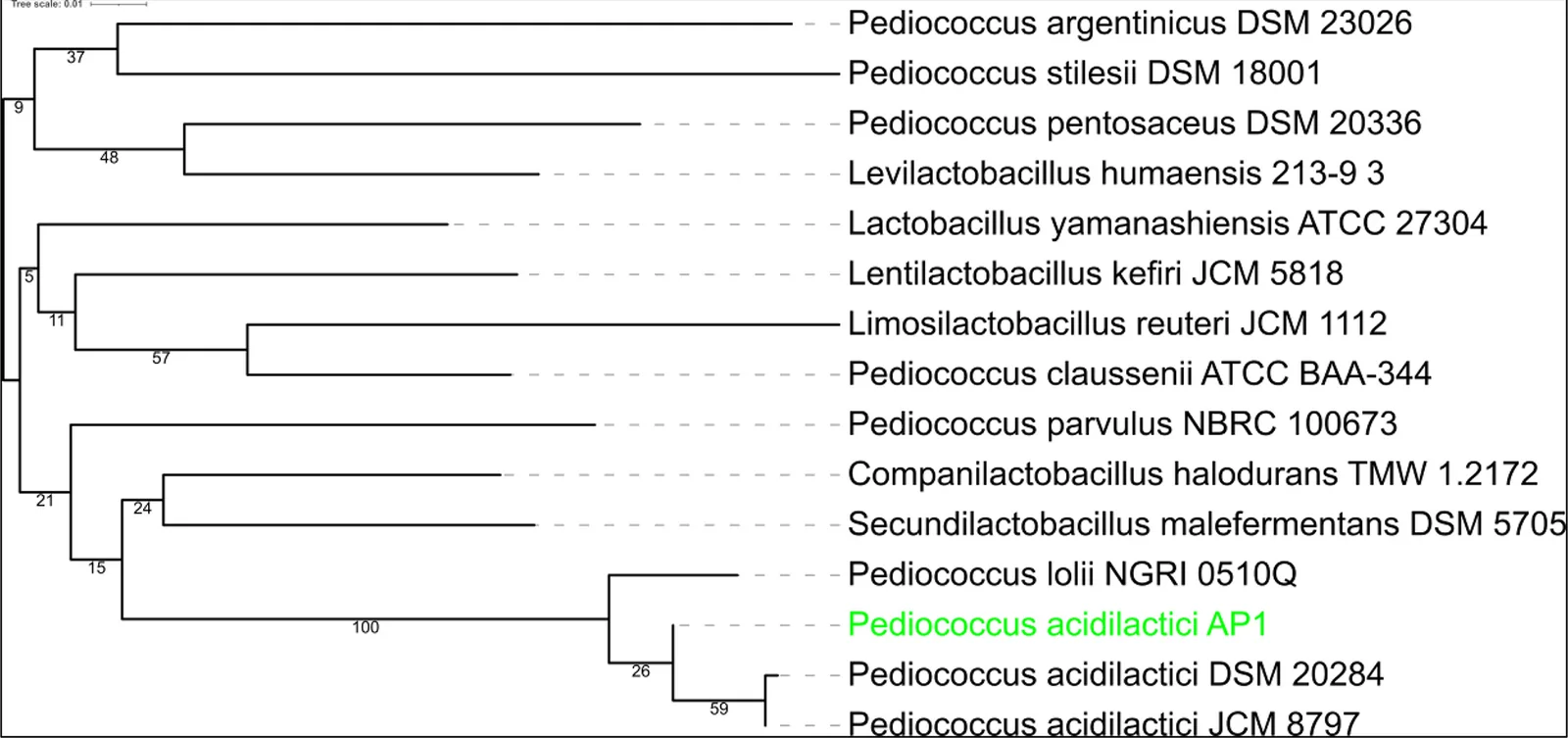
Genome-based phylogenetic placement of *Pediococcus acidilactici* strain AP1. The tree was inferred using FastME v2.1.6.1 from GBDP intergenomic distances calculated with formula d5 in TYGS. Branch support was estimated from 100 pseudo-bootstrap replicates, and the tree was midpoint-rooted. Numbers at the nodes indicate pseudo-bootstrap support values (%). Reference genomes were selected automatically by TYGS. The tree was visualized and annotated using the Interactive Tree of Life (iTOL) v7.6, and strain AP1 is highlighted in green.

De novo assembly generated a draft genome comprising 19 contigs with a total length of 2,015,983 bp, an N50 of 388,737 bp, an L50 of 2, a largest contig of 624,710 bp, and a GC content of 42.02%. CheckM2 estimated genome completeness and contamination at 99.99% and 0.5%, respectively, while BUSCO recovered 99.2% of the conserved bacterial single-copy orthologs. Table 1 summarizes the assembly statistics, genome-quality estimates, and structural annotation features.

**Table 1:** Assembly, quality-assessment, and annotation statistics of *Pediococcus acidilactici* strain AP1.

Feature	Value
Sequencing platform	Illumina MiSeq i100 Plus
Read configuration	Paired-end, 2 × 151 bp
Filtered paired-end read pairs	2,499,442
Read retention (%)	96.70
Genome size (bp)	2,015,983
GC content (%)	42.02
No. of contigs	19
Largest contig (bp)	624,710
N50 (bp)	388,737
L50	2
Mean sequencing depth (×)	163.2x
CheckM2 completeness (%)	99.99
CheckM2 contamination (%)	0.50
Complete BUSCOs (%)	99.2
Protein-coding sequences (CDSs)	1,897
tRNA genes	52
rRNA genes	4
tmRNA genes	1

PGAP annotation identified 1897 protein-coding sequences, 52 tRNA genes, four rRNA genes, and one tmRNA. Fig. 1 presents a circular visualization of the draft assembly generated using Proksee. The map displays coding sequences located on the forward and reverse strands, RNA genes, GC content, and GC skew. Because the assembly was generated from short-read sequencing data and consists of 19 contigs, Fig. 1 provides only a circular visualization of the draft assembly and should not be interpreted as evidence of a closed circular chromosome; the order and orientation of the contigs and continuity across unresolved repetitive regions remain undetermined.

Protein-coding sequence, tRNA, rRNA, and tmRNA counts were obtained from the Prokka v1.15.6 annotation. Assembly statistics were calculated using QUAST v5.3.0 with a minimum contig length of 500 bp. Genome completeness and contamination were assessed using BUSCO v6.0.0 and CheckM2 v1.1.0 respectively.

The taxonomic position of strain AP1 was confirmed using the Type Strain Genome Server (TYGS). Genome BLAST Distance Phylogeny (GBDP) analysis placed strain AP1 within the *Pediococcus acidilactici* lineage (Fig. 2). Strain AP1 exhibited a digital DNA–DNA hybridization (dDDH) value of 92.6% (95% confidence interval: 90.6–94.2%) with the *P. acidilactici* type strain DSM 20284T based on formula d4, substantially exceeding the 70% species delineation threshold. The small difference in genomic GC content (0.11%) further supported assigning strain AP1 to *P. acidilactici*.

To characterize the functional potential of strain AP1, the assembled genome was annotated using Prokka, RAST, eggNOG-mapper, and InterProScan. Integrated analysis of the annotation outputs identified genes involved in central carbohydrate and amino acid metabolism, membrane transport, cellular stress responses, and other conserved metabolic functions. The genome also contained genes putatively associated with traits relevant to probiotic candidate characterization, including acid and stress adaptation, peptide transport, exopolysaccharide biosynthesis, and cell-surface interactions. These genome-derived predictions are summarized in Table 2 and require phenotypic validation.

**Table 2:** Putative genome-encoded traits relevant to probiotic candidate characterization in *P. acidilactici* strain AP1.

Putative functional trait	Representative genomic features	Putative biological relevance
Glycolysis and lactate formation	*ldh, pyk, pfk, eno, gapA*	Carbohydrate utilization and lactate formation
Acid tolerance	*atpA, atpB, atpD, atpF, dnaK, groEL*	Putative contribution to adaptation under acidic conditions
Cellular stress response	*groEL, groES, dnaK, clpB, clpP, sodA*	Putative response to environmental stress
Exopolysaccharide biosynthesis	Putative glycosyltransferases and EPS-associated proteins	Potential exopolysaccharide biosynthesis
Peptide transport and proteolysis	*oppABCDF, pepN, pepC, pepX*	Peptide uptake and protein metabolism
Cell-surface-associated proteins	LPXTG-motif-containing proteins, *srtA*, and other predicted surface proteins	Potential cell-surface interaction
CRISPR–Cas system	*cas9, cas1, cas2, csn2* (Type IIA)	Adaptive defense against mobile genetic elements
Bile-associated adaptation	No identifiable *bsh* homolog	Bile tolerance cannot be inferred and requires phenotypic testing
Secondary-metabolite biosynthesis	One terpene-precursor region predicted by antiSMASH; one putative polyketide cluster predicted only by PRISM	Tentative biosynthetic potential; predicted products and biological functions remain unknown
Bacteriocin production	No bacteriocin or RiPP BGC detected by antiSMASH or PRISM	No bacteriocin-associated BGC identified under the applied analyses

Gene and biosynthetic gene cluster predictions indicate putative functional potential and do not demonstrate phenotypic activity. Experimental validation is required.

The annotated genome contained genes encoding enzymes involved in glycolysis and lactate formation, consistent with the fermentative metabolism characteristic of lactic acid bacteria. Genes encoding components of the F₀F₁-ATPase complex, molecular chaperones, and oxidative-stress response proteins were also identified, indicating the presence of conserved mechanisms potentially involved in adaptation to acidic and other environmental stress conditions. Multiple putative glycosyltransferases and polysaccharide-biosynthesis proteins potentially associated with exopolysaccharide formation were identified, together with peptidases and oligopeptide transport systems involved in peptide uptake and protein utilization.

A putative type II-A CRISPR–Cas system comprising *cas9, cas1, cas2*, and *csn2* was identified, suggesting a potential adaptive defense mechanism against bacteriophages and other mobile genetic elements. Putative LPXTG-motif-containing and other surface-associated proteins potentially involved in cell-surface interactions were also detected, although their biological functions require experimental validation.

Genome mining using antiSMASH identified one putative terpene-precursor region at positions 162,911–183,795 bp. PRISM independently predicted one putative polyketide biosynthetic cluster; however, no corresponding polyketide cluster was detected by antiSMASH. Therefore, the prediction of PRISM was considered tentative, and the products and biological relevance of both predicted regions remain unknown. No recognizable bacteriocin or RiPP biosynthetic gene cluster was detected by the applied genome-mining analyses. Likewise, no identifiable bile salt hydrolase (*bsh*) homolog was found, and therefore bile tolerance cannot be inferred from the genome annotation alone.

Taken together, the genome assembly, phylogenomic characterization, genome visualization, and functional annotation presented in this dataset provide a comprehensive genomic description of *P. acidilactici* strain AP1. The deposited genome sequence constitutes a useful resource for comparative genomic analyses of fish-associated *Pediococcus* strains and for future investigations of putative probiotic-relevant genomic characteristics.

## 4 Experimental Design, Materials and Methods

### 4.1 Bacterial isolation and genomic DNA extraction

*P. acidilactici* strain AP1 was isolated in April 2022 from the intestinal tract of a healthy common carp (*C. carpio*) obtained from a fish farm in the Tashkent region, Uzbekistan. The isolate was purified by repeated streaking on de Man–Rogosa–Sharpe (MRS) agar. The living strain AP1 has not been deposited in an international public culture collection. It is preserved at −80 °C in MRS broth supplemented with 20% (v/v) glycerol at the Institute of Microbiology, Academy of Sciences of the Republic of Uzbekistan.

For genomic DNA extraction, a single colony was inoculated into MRS broth and cultured at 37 °C for 48 h to obtain sufficient biomass. High-molecular-weight genomic DNA was extracted using the modified Marmur method described by Salvà-Serra et al. [8], with an additional modification. Briefly, bacterial cells were treated with lysozyme and RNase A, lysed with SDS, and extracted with chloroform–isoamyl alcohol (24:1, v/v). Following the addition of sodium acetate, DNA was precipitated with two volumes of 96% ethanol (aqueous phase: ethanol, 1:2, v/v) instead of isopropanol and incubated at -20 °C for 2 h. The precipitated DNA was subsequently recovered. DNA concentration was measured using a Qubit fluorometer (Thermo Fisher Scientific, USA), while DNA purity was assessed using a NanoPhotometer® N60 microvolume spectrophotometer (Implen, Germany). DNA integrity was evaluated by electrophoresis on a 1% agarose gel. The extracted DNA was subsequently used for whole-genome sequencing.

### 4.2 Whole-genome sequencing and genome assembly

Whole-genome sequencing was performed using the Illumina MiSeq i100 Plus System with paired-end 2 × 151 bp reads. Raw-read quality was evaluated using FastQC v0.12.1 [9]. Adapter sequences and low-quality bases were removed using fastp v1.1.0 [10]. Adapter sequences were automatically detected for the paired-end reads using the –detect_adapter_for_pe option. Low-quality bases were trimmed from both the 5′ and 3′ ends using a four-base sliding window and a mean Phred-quality threshold of Q20 (–cut_front, –cut_tail, –cut_window_size 4, and –cut_mean_quality 20). Bases with Phred scores of at least Q20 were considered qualified, and read pairs containing more than 40% unqualified bases were discarded. Reads shorter than 50 bp after trimming were also removed. Processing was performed using eight threads. The quality of the filtered reads was reassessed using FastQC.

Following quality filtering, 2,499,442 paired-end read pairs, corresponding to 96.70% of the initial read pairs, were retained for downstream analysis. All retained reads remained paired. De novo genome assembly was performed using SPAdes v4.2.0 [11] in –careful mode. The automatically selected k-mer sizes were 21, 33, 55, and 77. Read-error correction was performed using the integrated BayesHammer module, while Mismatch Corrector was enabled to reduce mismatches and short insertion/deletion errors in the resulting assembly.

Assembly statistics were calculated using QUAST v5.3.0 [12] in default mode with eight threads and a minimum contig-length threshold of 500 bp. Assembly completeness was assessed using BUSCO v6.0.0 [13] with the bacteria_odb10 lineage dataset. Genome completeness and contamination were independently estimated using CheckM2 v1.1.0 through the Galaxy platform (Galaxy wrapper version 1.0.2+galaxy2) [14].

### 4.3 Genome annotation, functional analysis, and phylogenomics

Taxonomic classification was initially performed using Kraken2 v2.17.1 with the Kraken2 Standard database (8 GB; build date: 12 January 2024) [15]. Genome-based taxonomic identification was subsequently performed using TYGS [16]. For phylogenomic inference, pairwise comparisons among the selected genomes were performed using the Genome BLAST Distance Phylogeny (GBDP) approach under the “trimming” algorithm and distance formula d5. One hundred distance replicates were calculated for each comparison. Digital DNA–DNA hybridization (dDDH) values and their 95% confidence intervals were calculated using the recommended settings of GGDC 4.0. Formula d4 was used for species-level interpretation because it is robust against differences in genome length and the incompleteness of draft genome assemblies.

The resulting intergenomic distances were used to infer a balanced minimum-evolution tree using FastME v2.1.6.1 with subtree pruning and regrafting (SPR) post-processing. Branch support was inferred from 100 GBDP pseudo-bootstrap replicates, and the tree was midpoint-rooted. The resulting tree was exported in Newick format and visualized using the Interactive Tree of Life (iTOL) v7.6.

Structural genome annotation was performed using the NCBI Prokaryotic Genome Annotation Pipeline (PGAP) and Prokka v1.15.6 [17]. Functional annotation was independently performed using the RAST server [18]. Predicted protein sequences were further analyzed using eggNOG-mapper v2.1.13 [19] and InterProScan v5.77-108.0 through the Galaxy platform to identify orthologous groups, conserved protein domains, protein families, and putative biological functions [20].

Functional annotations generated by the different platforms were manually cross-compared using gene names, orthologous-group assignments, and predicted protein domains. This analysis was used to identify putative genome-encoded traits relevant to probiotic characterization, including genes potentially associated with glycolysis and lactate formation, acid-stress adaptation, cellular stress responses, exopolysaccharide biosynthesis, peptide transport and proteolysis, and cell-surface interactions.

Biosynthetic gene clusters were investigated using antiSMASH v8.0.4 and PRISM v4.4.5 with default parameters. Predictions identified by only one genome-mining platform were considered tentative. CRISPR arrays and associated Cas gene clusters were identified using CRISPRCasFinder [17] with default parameters, and the resulting data were incorporated into the genome annotation dataset. Genome visualization was generated using Proksee v1.1.0 [18] based on the annotated draft genome assembly.

## Limitations

The genome sequence of strain AP1 represents a draft assembly generated exclusively from short-read Illumina sequencing data. Consequently, repetitive genomic regions, structural variants, plasmids, and other extrachromosomal elements may not have been fully resolved. The functional traits and biosynthetic gene clusters described in this dataset were predicted computationally and do not demonstrate gene expression, metabolite production, or phenotypic probiotic activity. In particular, the putative polyketide biosynthetic gene cluster predicted only by PRISM should be considered tentative because it was not independently detected by antiSMASH. Long-read sequencing would be required to obtain a closed genome, whereas experimental validation would be necessary to confirm the predicted functional characteristics and biosynthetic potential.

## Ethics Statement

*Pediococcus acidilactici* strain AP1 was isolated from the intestinal tract of a common carp (*Cyprinus carpio*) obtained from a commercial fish farm in the Tashkent region, Uzbekistan. The fish was slaughtered as part of routine commercial farm operations and was not killed specifically for this work. No experiments involving live animals were conducted.

## Credit Author Statement

**Elova Nilufar:** Conceptualization, Data curation, Formal analysis, Methodology, Validation, Writing – original draft; **Bobokulov Murodali:** Formal analysis, Methodology, Data curation and Visualization. **Bekmurodova Gullola:** Conceptualization, Investigation, Writing – review & editing; **Amirsaidova Dildora:** Data curation, Formal analysis; **Xidirova Mastura:** Resources, Writing – original draft. **Yodgorova Farangiz:** Conceptualization, Investigation. **Kobilov Fazliddin:** Conceptualization, Investigation, Project administration, Supervision, Writing – review & editing. **Miralimova Shakhlo:** Funding acquisition, Project administration, Resources, Supervision.

## Funding

This work was supported by institutional baseline funding provided to the Institute of Microbiology, Academy of Sciences of the Republic of Uzbekistan.

## Data Availability

doi:10.17632/wjdntc39sp.1
